# Reflective and Transmissive Off-Axis Sub-THz Beam Shaping Using Passive Complementary Split-Ring Resonators

**DOI:** 10.3390/s26217013

**Published:** 2026-11-06

**Authors:** Ernestas Nacius, Vladislovas Čižas, Kasparas Stanaitis, Ignas Grigelionis, Linas Minkevičius

**Affiliations:** Department of Optoelectronics, Center for Physical Sciences and Technology, Saulėtekio ave. 3, LT–10257 Vilnius, Lithuania; ernestas.nacius@ftmc.lt (E.N.); vladislovas.cizas@ftmc.lt (V.Č.); kasparas.stanaitis@ftmc.lt (K.S.); ignas.grigelionis@ftmc.lt (I.G.)

**Keywords:** metasurface, terahertz, dual-mode operation, reflector, beam-shaping, Bessel beam, Gaussian beam

## Abstract

Terahertz and sub-terahertz links offer wide bandwidths for future wireless communication, but practical deployment is limited by high propagation loss, atmospheric absorption, and sensitivity to blockage in non-line-of-sight environments. Here, we demonstrate ultrathin, passive full-space beam-shaping elements based on complementary split-ring resonators laser-cut into stainless steel foil. By encoding the target phase profile in the spatial arrangement of identical complementary split-ring resonator meta-atoms, which act as a binary amplitude-type zone plate, we shape the wavefront simultaneously in transmission and reflection and form foci on both sides of a single element. We show that off-axis routing (e.g., toward $45^{\circ }$) is achieved via pattern arrangement without re-optimizing the complementary split-ring resonator meta-atoms for each geometry, providing a straightforward route to dual-channel routing. As a proof of concept, we transform a collimated incident field into Gaussian and Bessel-type beams and compare their propagation characteristics with more classical Fresnel-lens-like reflectors. These results suggest a potential low-profile approach to passive sub-THz beam control, which may support alignment-tolerant operation and could, in future work, be extended toward non-line-of-sight channel conditioning and compact multi-focus beam shaping.

## 1. Introduction

The terahertz (THz) and sub-THz spectral regions (0.1–10 THz) provide large underutilized bandwidth resources that can enable ultra-high-capacity wireless links and sensing systems [1,2]. At the same time, THz propagation is fundamentally constrained by high free-space path loss and frequency-selective atmospheric absorption, particularly due to water vapor [3]. These constraints are widely recognized as central obstacles for practical THz networking and motivate highly directive, wavefront-engineered front-ends [4,5]. Another key deployment challenge is robustness under blockage and non-line-of-sight (NLOS) conditions. At low THz frequencies, links can be disrupted by common obstacles and by rapid channel fluctuations. Moreover, reflections and scattering from materials and rough surfaces can dominate viable NLOS paths [6,7]. Recent measurements show that even diffuse scattering can support data links above 100 GHz, highlighting both the difficulty and opportunity of engineering controllable NLOS routes [8].

The advanced beam-shaping can mitigate these transmission impairments. Currently, THz radiation is predominantly manipulated by gold-coated surfaces (i.e., parabolic mirrors, etc.) or reflectarray antennas for reflective beam-shaping, as well as transmissive beam-shaping components including dielectric and Fresnel lenses, fabricated from silicon or other high-permittivity materials [9,10,11]. Reflective beam-shapers generally offer higher efficiency, and they can be made reconfigurable for tunable beam steering in practical communication links [7,12,13]. However, the gold-coated reflective solutions are usually bulky, while the reflectarray antennas are comparably harder to fabricate. Transmissive beam-shapers not only typically suffer from increased losses associated with absorption and Fresnel reflections, but also require extremely high fabrication accuracy of the surface to achieve precise phase control [10,14,15]. Metasurfaces have emerged as a powerful approach for compact THz beam and wavefront control through engineered phase discontinuities [16,17]. Another advantage of this approach is its unique control over the beam’s polarization state [18]. This capability is particularly relevant for beams intended for data transmission, as it enables the possibility for multi-channel communication using distinct polarization states [19]. Beyond beam focusing, metasurfaces now address a broad range of functions, including intensity-asymmetric wavefront shaping with nonlocal meta-lenses [20], secure wireless communication enabled by space–time-coding metasurfaces [21], nanoscale alignment based on non-local bound states in the continuum [22], and adaptive light-field imaging with varifocal metalens arrays [23]. These developments show that a single patterned surface can take over functions that otherwise require bulky or multi-component optics, which also motivates compact passive metasurface elements for sub-THz links. Nevertheless, practical high-efficiency designs often require precise sub-wavelength patterning and frequently employ multilayer or multi-material unit cells, complicating fabrication and scalability [24,25,26,27].

In this work, we investigate dual-mode off-axis THz beam-shaping structures, operating at 253 GHz due to the availability of a specific continuous-wave source, as well as because this frequency falls in the atmospheric transmission window, suitable for future communication applications [3]. The proposed thin metallic foil-based elements are demonstrated to enable simultaneous wavefront manipulation in both transmission and reflection modes. Two distinct approaches are analyzed: a beam-shaping structure, based on an elliptic Fresnel zone plate, and a metamaterial-based solution. The Fresnel-type solution is implemented in a $45^{\circ }$ angle of incidence (*AoI*) arrangement, whereas the metasurface-based approach employs an alternative geometry with normal incidence ($AoI=0^{\circ }$) onto the structure. The latter configuration is realized using a metasurface composed of complementary split-ring resonator (CSRR) meta-atoms. The complementary split-ring resonators are sub-wavelength slot-type metamaterial structures cut-out in a conductive material plane that behave as LC resonators, where electromagnetic excitation induces coupled currents in the split-ring, producing a sharp resonance typically associated with effective negative permittivity [28,29,30,31,32]. In general, varying the geometrical parameters of the meta-atoms controls the local spectral and phase shift parameters. In this research, a single meta-atom geometry is used, so each element acts as a binary amplitude-type zone plate: zones filled with CSRRs transmit and reflect at resonance, while the unpatterned foil reflects. The transmitted and reflected foci therefore form a mirror-symmetric pair on opposite sides of the element and lie outside the incident beam, which is not attainable with the Fresnel-type dual-mode configuration. CSRR metalenses laser-cut into stainless steel foil were designed as on-axis Fresnel zone plates and employed as a focusing solution in a dual-mode THz homodyne imaging system operating simultaneously in transmission and reflection, which have already been presented in previous works [33,34]. In contrast, in the presented work, a single metasurface-based element, arranged as a binary zone plate, shapes the beam off-axis at normal incidence, forming Gaussian or Bessel-type foci on both of its sides, outside the incident beam. As a proof-of-concept demonstration, the proposed structures were designed to transform an incident collimated beam into Gaussian and Bessel-type beam profiles [35,36]. Both dual-mode approaches are expected to provide several advantages for THz data transmission systems. First, two independent sources or detectors may be positioned at the corresponding focal regions of the structure, enabling the realization of dual-channel multiple-input multiple-output (MIMO) architectures [37,38]. Second, the proposed off-axis beam-shaping solutions may contribute to mitigating the limitations associated with NLOS communication scenarios [39,40].

## 2. Materials and Methods

**Figure 1:**
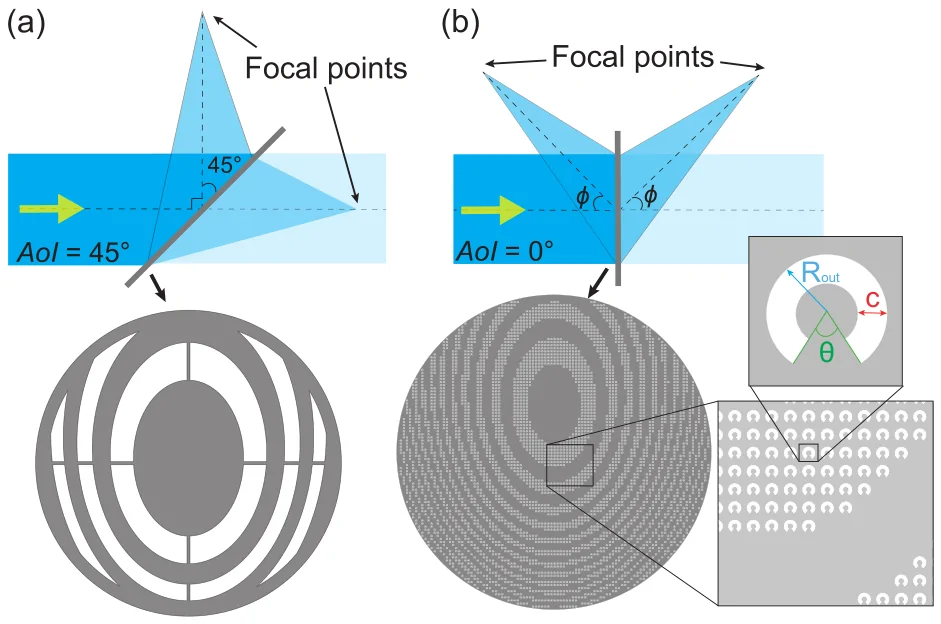
Principal schematics of the investigated beam-shaping elements demonstrating the operation principle of (**a**) a Fresnel lens-based binary reflector realized using elliptical beam-shaping zones in the $AoI=45^{\circ }$ configuration; (**b**) a metasurface-based element designed for operation at $AoI=0^{\circ }$. Beam tilting angle is denoted as ϕ; in both configurations it is measured between the element normal and the direction from the element center towards the focus. The insets illustrate the meta-atoms comprising the metasurface structure, together with the three resonance- and phase-defining geometrical parameters of the complementary split-ring resonator meta-atoms: the outer radius $R_{\mathrm{out}}$, split-ring thickness *c*, and conductive gap angle θ. Yellow arrows indicate the direction of the incident radiation.

For Gaussian and Bessel beams, the phase surface equations corresponding to on-axis beamforming are well established. Therefore, the realization of off-axis beamforming requires only minor modifications to account for the altered optical path difference. We implemented a Fresnel lens-based beam-shaping structure at an $AoI=45^{\circ }$ [see Figure 1a], a standard arrangement that simplifies optical layout and alignment. The resulting structure can be described as follows [41]:

(1) $${\left(\frac{x}{a_{n}\cos\phi }\right)}^{2}+{\left(\frac{y-b_{n}}{a_{n}}\right)}^{2}=1.$$

Here, $a_{n}=\sqrt{n\lambda \left(f\cos^{2}\phi +\frac{n\lambda }{4}\right)}/\cos^{2}\phi$, where λ denotes the wavelength, *n* represents the zone boundary index, *f* is a focal distance, and $b_{n}=n\lambda \sin\phi /2\cos^{2}\phi$. In this configuration, the incident radiation is redirected by an angle ϕ, and the element is designed to form a focused beam at the same angle ϕ. The dual-mode elliptical beam-shaping structure is realized by forming elliptical zones of reflective material separated by radiation-transmitting air gaps. However, as evident from the principal schematic, for the majority of the investigated ϕ values, the transmitted beam is formed within the spatial region of the incident beam. This represents a significant limitation of the proposed configuration, as it is expected to substantially increase the noise level in the transmitted beam component.

The aforementioned limitation could, in principle, be mitigated by employing the $AoI=0^{\circ }$ configuration. Such an approach would enable symmetric off-axis beamforming, as illustrated in Figure 1b. However, it can be readily observed that the radii of the Fresnel zones are substantially reduced in this configuration. Considering that the Fresnel lens approach requires every second sub-zone to be completely removed in the case of binary discretization K=2 (metal and cutout zones), the resulting structure would become mechanically fragile and insufficiently stable. Consequently, its beam-shaping performance and practical implementation reliability would be significantly degraded. Therefore, the $AoI=0^{\circ }$ approach is considered more suitable for the metasurface-based realizations. The case of $AoI=0^{\circ }$ Gaussian beam-forming is described using the non-paraxial lens equation for a hyperbolic surface, augmented by lateral offset coefficients computed for the desired beam-steering angle and focal length. For a focusing reflector, the phase surface is expressed as follows [42]:

(2) $$\varphi \left(x,y\right)=k\sqrt{{\left(x-f_{x}\right)}^{2}+y^{2}+f_{z}^{2}},$$

where *k* denotes the wavenumber, $f_{x}$ is the lateral offset of the focus from the optical axis, and $f_{z}$ is the distance of the focus along the optical axis (*z*). With the tilt angle ϕ measured from the optical axis [see Figure 1b], the focus lies at $\left(f_{x},0,f_{z}\right)$ with $f_{z}=f_{x}/\tan\phi$, i.e., at a distance $f_{x}/\sin\phi$ from the element center.

We characterize the discretization by the number of levels *K*, counting the unpatterned foil as one level. Both elements studied in this work are therefore binary (K=2): the Fresnel-type element alternates metal and air zones, whereas the metasurface alternates CSRR-filled and unpatterned zones. Contrary to the Fresnel lens-based approach, higher-level discretization (K>2) is available for the metasurface-based structures by using several meta-atom geometries with different resonance phases, i.e., adjusting the geometrical parameters of the meta-atoms employed in each of the sub-zones, ensuring correct relative phase-shift across the sub-zones. Nevertheless, it is important to emphasize that previous studies on on-axis beamforming configurations have demonstrated that increasing the discretization level yields only marginal theoretical performance improvements. Under practical conditions, such improvements may become negligible due to fabrication imperfections of the meta-atoms, which can alter the intended relative phase response. Consequently, discretization levels up to K=4 were identified as the most optimal compromise between theoretical performance and fabrication tolerance [43]. In the present proof-of-concept study, however, a binary design (K=2) was selected, in which a single meta-atom geometry fills every second sub-zone of the structure. With a single geometry, the local phase cannot be varied across the element; instead, the target phase profile φ(x,y) is encoded in the positions of the zones. Meta-atoms are placed where cosφ(x,y)≥0, and the foil is left intact elsewhere, so that the zone boundaries follow contours of the target phase, as in a Fresnel zone plate or a binary computer-generated hologram. The first diffraction order then reproduces the target wavefront. The modulation itself is therefore of amplitude type. The CSRR-filled zones transmit at resonance, whereas the unpatterned foil reflects. Such an approach eliminates errors associated with relative phase-shift deviations, since the relative phase response between different meta-atom geometries becomes irrelevant. It is worth noting that, in this case, the beam-shaping performance in transmission is determined solely by the geometry of a single meta-atom, whereas in reflection it is determined by the interference between the fields re-radiated by the meta-atom-patterned regions and those reflected by the unpatterned zones. This approach offers additional control over the reflected beam: filling the unpatterned zones with non-resonant meta-atoms would reduce their contribution to the reflected field.

Another advantage of the proposed structures is their comparatively straightforward adaptation to different resonant frequencies and/or beam types. In contrast to many currently employed beam-shaping solutions, the proposed approaches do not require modifications of the fabrication process parameters when the beam-shaping characteristics are altered. For example, changing the type of generated beam only requires modification of the contour profile in the Fresnel lens-based design, whereas in the metasurface-based approach only the spatial arrangement of the meta-atoms must be adjusted. This enables the construction of more complex fields while the manufacturing parameters themselves remain unchanged, considerably simplifying the adaptation procedure. As a demonstration, the Bessel beam can be created by modifying a conical phase surface:

(3) $$\varphi \left(x,y\right)=k\left(\sqrt{{\left(x-f_{x}\right)}^{2}+y^{2}+f_{z}^{2}}+\sqrt{\left(x^{2}+y^{2}\right)}\sin\beta \right).$$

Here, β denotes the Bessel beam convergence angle. Having defined the phase surfaces corresponding to both Gaussian and Bessel beam-forming configurations, the meta-atom distribution was subsequently determined, enabling the finalization of the structure for numerical simulation and fabrication.

The geometrical parameters of the meta-atoms are optimized using a precomputed database of meta-atoms, where three main geometrical parameters of the meta-atom are considered: the conductive gap angle θ, the outer radius $R_{\mathrm{out}}$, and the split-ring thickness *c*. By adjusting these parameters, the desired resonant frequency and corresponding phase-shifted response can be achieved. Although several analytical models exist that relate geometrical features to spectral characteristics [44,45], a numerical database-based approach was found to provide the most precise accuracy; therefore, the optimized meta-atom parameters of c=90 µm, $R_{\mathrm{out}}=170$ µm, and $\theta =65^{\circ }$ were used in this work.

The optimized meta-atoms were subsequently arranged into a metasurface-based zone plate with a diameter of D=50.8 mm. The focal points for both Gaussian and Bessel beams were defined to be located at a distance of $f_{x}=5$ cm from the optical axis. This choice ensures that, independent of the focusing angle (or focal depth $f_{z}$), the focal region does not overlap with the spatial extent of the incident beam. A Bessel beam with a convergence angle of $\beta =10^{\circ }$ was employed, consistent with our previous work [46].

**Figure 2:**
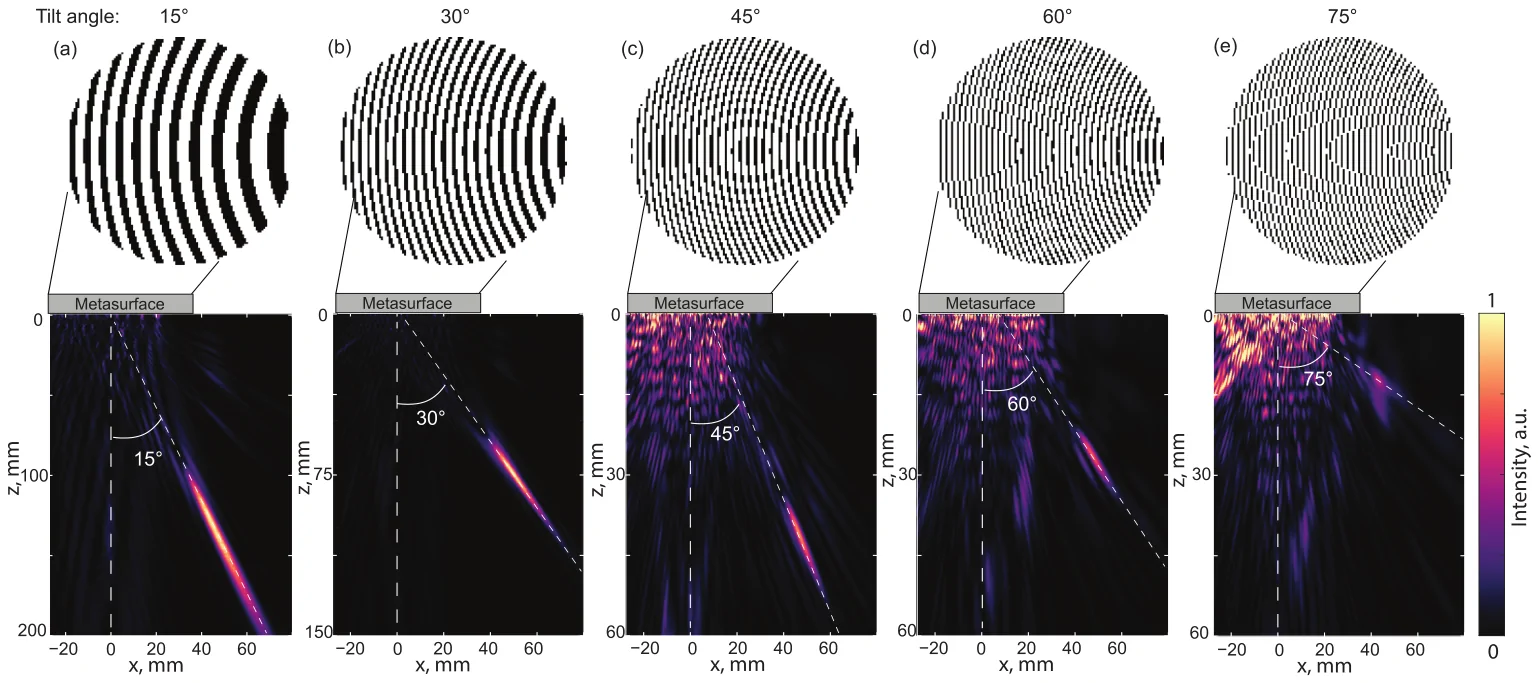
Designs (top row) and simulated intensity distributions in the xz plane (bottom row) of off-axis Gaussian beam-forming meta-elements with focusing angles ranging from $15^{\circ }$ to $75^{\circ }$ in increments of $15^{\circ }$. In the metasurface masks, dark regions correspond to meta-atoms, whereas white regions indicate the unmodified metal foil. Note that the scale of the *z* axis differs between panels (**a**–**e**), as the axial focal distance $f_{z}=f_{x}/\tan\phi$ decreases with the tilt angle. Coarse dashed lines denote the optical axis of the system, while fine dashed lines indicate the tilted axis of the focal line, forming the angle ϕ with the optical axis. The intensity maps are normalized to their respective maximum values. Intensity as well as the integrated intensity values can be found in the Supplementary Material, Table S1.

Prior to fabrication, numerical simulation of the designed beam-shaping structures was carried out to ensure the correctness of the design. The simulations were performed using CST Studio Suite 2026 (Dassault Systèmes). A range of beam tilting angles was investigated, spanning from $15^{\circ }$ to $75^{\circ }$, as illustrated in Figure 2. For all cases, the angle of incidence was $AoI=0^{\circ }$, Gaussian focusing patterns were selected, and only transmission fields were investigated to avoid strong reflections from surfaces and interference with the incoming beam. Several important features are visible from the simulation results. It is clearly visible that the focal intensity diminishes with increasing deflection angle. The intensity integrated over the half-maximum area of the focus in the xz plane, which accounts for the focal size, decreases monotonically: 559, 277, 4.4, 3.2 and 1.9 (V/m)^2^ mm^2^ for $\phi =15^{\circ }$, $30^{\circ }$, $45^{\circ }$, $60^{\circ }$ and $75^{\circ }$ (see the Supplementary Material, Figure S1 and Table S1). The steep drop for $\phi \geq 45^{\circ }$ coincides with the sub-zones becoming about two meta-atoms wide or narrower. This is explained by two different effects. First of all, one should note that the sizes of the sub-zones as depicted in Figure 2 decrease in size with an increase in deviation angle. This is of extreme importance, as the meta-atom spectral characteristics have been acquired under infinite array conditions featured by a period of 2.5 times the outer radius of the meta-atom. It is very well known that the resonance of the meta-atom depends on the period of the mesh; thus, with the decrease in the sub-zones, an increasing reduction in focal intensity may be observed due to the resonance peak shift, occurring due to the decreased inter-meta-atom interaction [47]. Furthermore, a decrease in intensity is supported by the dipolar angular momentum distribution [48]. Still, the possibility of deflection at relatively large tilt angles [e.g., $60^{\circ }$–$75^{\circ }$ as depicted in Figure 2d,e] is revealed. More information on the profiles is presented in the Supplementary Material [32,33,49,50,51]. Based on the presented simulations, the study was further continued by selecting metasurface-based elements designed to tilt the beam at an angle of $45^{\circ }$, with extensive investigation of operation in the reflection regime.

**Figure 3:**
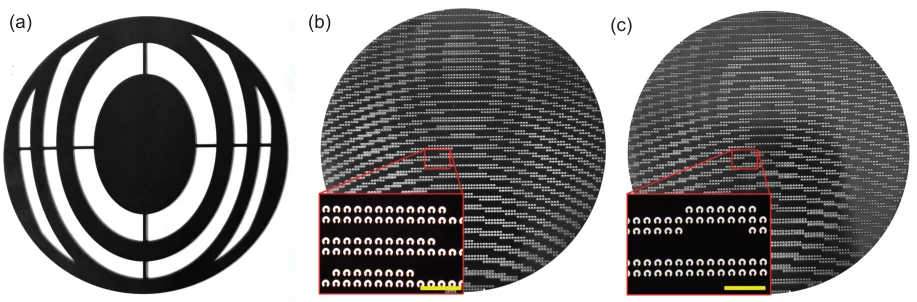
Images of manufactured elements from stainless steel foil: (**a**) Fresnel lens-based elliptical beam-shaping structure designed with a phase profile corresponding to $\phi =45^{\circ }$ and a focal length of f=70 mm; (**b**) Gaussian beam-focusing metasurface-based element; (**c**) Bessel beam-shaping metasurface-based element. The insets reveal transmission light microscope (“Hirox KH-7700”, Hirox (Tokyo, Japan)) Images of the complementary split-ring resonator patterns. All three elements are 50.8 mm in diameter. The Gaussian and Bessel beam-shaping metasurface elements were made out of 25 µm thick foil, while the Fresnel reflector was cut out from 300 µm thick foil for better rigidity. The yellow scale bars shown in the insets are 2 mm.

Several manufactured structures are revealed in Figure 3. The manufacturing procedure of the elements and their beam-shaping characteristics have been extensively described and depicted in the Supplementary Material, Figures S2 and S3. The main experimental work consisted of a THz detector mounted on motorized three-axis (*xyz*) translation stages with an attached silicon spherical lens for maximum collection of radiation. The laser-manufactured structures were illuminated by a collimated 253 GHz continuous-wave source providing a Gaussian input beam shape. The detector, mounted on the translation stages, was used to scan intensity in both transmission and reflection planes, perpendicular to the optical axis.

## 3. Results and Discussion

**Figure 4:**
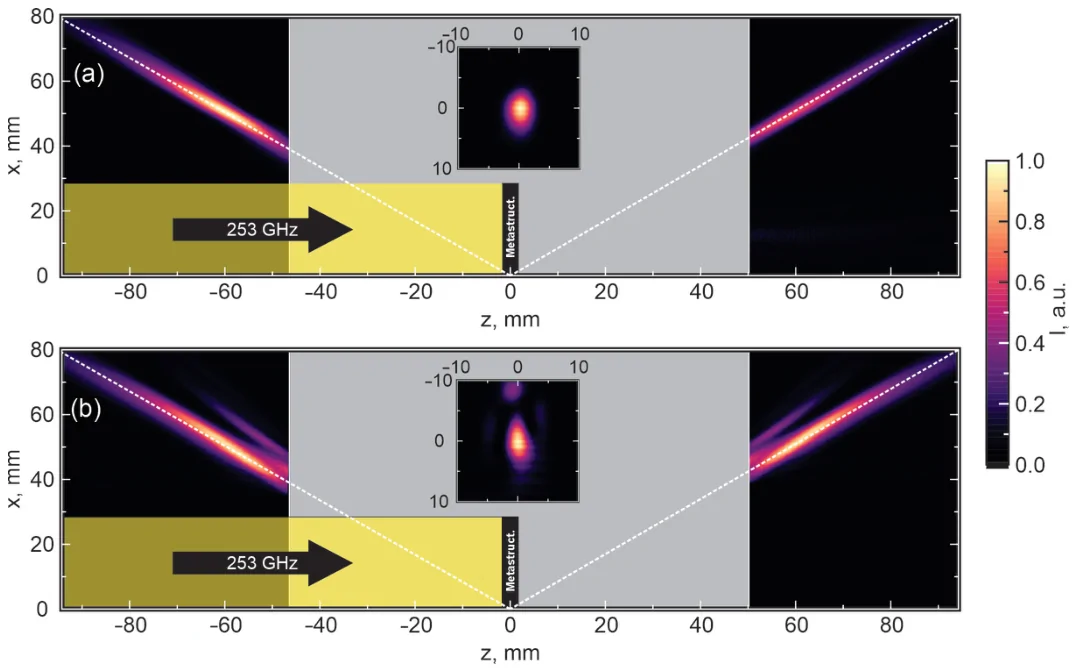
Experimentally obtained intensity distributions of the designed meta-elements generating Gaussian (**a**) and Bessel (**b**) beams. Lighter colors indicate regions of higher intensity, whereas darker colors correspond to lower intensity. The yellow region and black arrow denote the incident radiation of 253 GHz. The black squares labeled “Metastruct.” indicate the position of the metasurface-based beam-shaping structure. Gray regions correspond to areas not scanned due to experimental setup limitations. The central insets show the transverse intensity profiles measured normal to the beam-propagation axis (white dashed line). Both intensity maps are normalized to their respective maximum values.

Experimental intensity distributions of the generated Gaussian and Bessel beams are presented in Figure 4. Beam-shaping performance was evaluated by measuring the FWHM and the depth of focus (DOF) of the generated beams. The DOF was determined from a one-dimensional intensity profile extracted from the yz scan along a line extending from the center of the metasurface to the location of maximum intensity of the formed beam, i.e., along the tilted focal line, as the length over which the intensity exceeds 50 % of its maximum. The FWHM was evaluated from a transverse one-dimensional intensity profile extracted from the xy scan, which is perpendicular to the focal line, at the position of peak intensity. In particular, this includes coordinate scaling according to the tilting of the profile (a scaling factor of cosϕ was applied to the *y* coordinate in the xy plane, and a factor of 1/cosϕ was applied to the *z* coordinate in the yz plane). The same criteria were applied to the simulated fields. The measured parameters together with the corresponding simulation results are summarized in the Supplementary Material, Figure S7. The experimental results reveal an FWHM of 3.5 mm and 3.0 mm in transmission and 3.5 mm and 3.8 mm in reflection, respectively, for Gaussian and Bessel structures. Experimental results are expected to be larger than the simulated values of 2.6 mm and 2.8 mm in transmission and 2.4 mm and 3.2 mm in reflection, respectively, for Gaussian and Bessel structures. For the Bessel beam-forming metasurface, the experimentally measured DOF of 30.4 mm in transmission and 31.1 mm in reflection closely matches the simulated value of 33.3 mm and 33.8 mm. In contrast, a substantial discrepancy between the experimental and simulated DOF values was observed for the Gaussian beam-forming metasurface. Numerical simulations predict a DOF of 17.9 mm in transmission and 19.6 mm in reflection, whereas the experimentally measured values reach 31.5 mm and 35.0 mm. The estimated focus gain for the Gaussian beam-forming structure was found to be about 5.0 and 3.3 in transmission and reflection, respectively, if the average power at the Bessel-like zero-order focal line for each case is compared to the average value of incoming beam power at its $1/e^{2}$ area. In terms of total power, this structure directed approximately 26% of the incident power into the transmitted focal line and 16% into the reflected one. For the case of the Bessel beam-forming structure, the metalens provides a weaker gain of about 1.5–1.6 in transmission and at least 1.5 in reflection, corresponding to approximately 11% and 5% of the incident power, respectively. In all of the cases, a pronounced deviation, which was not observed for previously reported on-axis beam-shaping designs, can be explained by considering the effective excitation area of the metasurface.

Under ideal conditions, metasurface designs are typically analyzed assuming plane-wave illumination, for which each meta-atom is considered to be equally excited by the plane wave of the same phase regardless of its position within the structure. However, in the experimental setup employed in this work, the metasurfaces are illuminated by a Gaussian-like beam with a finite spatial extent (FWHM of 12.3 mm and 15.8 mm in *x* and *y* axes, respectively) rather than by an ideal plane wave (see the Supplementary Material, Figure S5). Consequently, the contribution of individual meta-atoms becomes nonuniform, with the central region of the metasurface receiving significantly higher excitation than its outer regions. Inspection of the meta-atom designs shown in Figure 3b,c reveals the similarities between the Gaussian and Bessel beam-forming structures at the central area. Under Gaussian-beam illumination, the increased weighting of these central regions enhances their influence on the generated beam. For the asymmetric beam-shaping structure designs, this effect effectively shifts the expected Gaussian beam characteristics toward those of a Bessel beam, resulting in a substantial increase in the measured DOF.

Although the obtained results provide grounds for further optimization of the metastructure design, particularly with respect to adaptation to different excitation beam profiles, such sensitivity is not expected to pose a significant limitation in practical NLOS-like applications. In realistic deployment scenarios, the substantially larger propagation distances between the source and the metasurface are expected to ensure a nearly planar incident wavefront, thereby reducing the impact of nonuniform illumination. Furthermore, when considering the operating regime in which two sources are positioned at the reflected and transmitted foci of the metasurface, the effective DOF is expected to approach the values predicted by numerical simulations. Conversely, the extended DOF provided by both of the structures is anticipated to relax alignment tolerances and enhance robustness to the positioning errors. This characteristic may also facilitate the analysis of volumetric or optically thick objects, thereby extending the applicability of the proposed solution to be employed in a broader range of imaging and sensing scenarios [52,53].

## 4. Conclusions

In summary, we experimentally verified metasurface-based THz beam-shaping elements, capable of simultaneously working in both transmission and reflection modes. Experimental and numerical results validate the ability of the proposed structures to generate well-defined Gaussian and Bessel beam profiles with controlled off-axis propagation, demonstrating beam steering over a wide angular range. Observed deviations between the experimental and simulated results are primarily attributed to the nonuniform Gaussian illumination of the finite aperture, rather than the ideal plane-wave excitation assumed in the simulations, providing a basis for future research and design optimization. The demonstrated structures achieve beam widths on the order of a few wavelengths and sustained propagation characteristics in both reflective and transmissive modes, indicating their suitability for dual-channel operation.

The presented approach shows potential for next-generation communication systems, particularly for addressing non-line-of-sight propagation and, through symmetric beam formation in transmission and reflection modes, for the future physical realization of multiple-input multiple-output (MIMO) systems. Furthermore, the presented approach may support future developments such as polarization control via structural rotation and beam steering through mechanical deformation (e.g., bending) [32]. Beyond communication systems, the demonstrated concept may also find applications in dual-sample imaging, enabling real-time imaging and comparison with a reference sample without modification of the experimental setup. 

## Data Availability

The data that support the findings of this study are available from the corresponding author upon reasonable request.

## Supplementary Materials

The following supporting information can be downloaded at https://www.mdpi.com/article/10.3390/s26217013/s1. Figure S1: Axial intensity profiles (a) along the focal line and (b) along the perpendicular axis for the Gaussian beam shaping structures, designed to focus radiation at different tilting angles from $15^{\circ }$ to $75^{\circ }$. The intensity curves are normalized to their respective maximum values; Table S1: Parameters of the simulated transmitted focal zones for tilt angles from $15^{\circ }$ to $75^{\circ }$: depth of focus (DOF, full width at half maximum of the intensity along the tilted focal line), FWHM perpendicular to the focal line, peak intensity $I_{\mathrm{peak}}={\left|E\right|}^{2}$ for an incident plane-wave amplitude of 1 V/m, area $A_{1/2}$ enclosed by the half-maximum contour of the focus in the xz plane, and intensity integrated over this area. Values in brackets are normalized to the wavelength (λ≈1.19 mm); Figure S2: Power reflection and transmission (top row) and the corresponding phase spectra (bottom row) of the metaatom with the geometrical parameters used in the manuscript, for y-polarized incidence. Left column: co-polarized response (YY); right column: cross-polarized response (XY). Subscripts denote reflection (11) or transmission (21), and superscripts denote the output and input polarization, respectively; Figure S3: Microscope images under backlight illumination of complementary split-ring resonators, cut out by using laser ablation through 25 µm thick steel foil. (a) shows a single complementary split-ring resonator with measured dimensions: inner and outer radii, cut-out angle, and ring thickness. (b) shows array of such resonators in reduced magnification, displaying consistency of fabricated structures; Figure S4: (a) The experimental scheme for the investigation of the dual-mode off-axis beam-shapers: PG – pulse generator "Agilent 33519B", LA – Lock-in amplifier "7265 DSP Signal Recovery", S – linear polarization source "CMOS Colpitts", emitting 78 µW power 253 GHz radiation, PM - parabolic mirror of focal length f=15 cm, PC – computer used for process automatization and a 90 nm CMOS TeraFET detector synchronous scanning in xyz axes (*y* – LTS300C, xz – DDS300, both products of *ThorLabs, Inc.*), D1 and D2 represent two different positions in which a THz detector was placed during scans. AB - radiation absorber, which ensures no back-reflections and detector biases occur. (b) Angular dependence of the normalized focal spot signal measured with the lens-coupled CMOS TeraFET detector module, where black markers indicate experiment, red curve is exponential fit. The dashed horizontal line indicates the $1/e^{2}$ level and the vertical dashed line marks the corresponding characteristic angle ($\alpha_{1/e^{2}}=15.8^{\circ }$). The insets show representative measured focal spot intensity maps at selected tilt angles; Figure S5: Intensity profile, acquired after the parabolic mirror in the setup. Note the non-uniformity in the profile, explaining discrepancies between the simulated and experimentally achieved results; Figure S6: Characterization of the Fresnel lens-based beam shaper operating in reflection and transmission modes. Figures on the left [(a) and (c)] show the intensity distributions in the xz plane corresponding to the focal region of the structure for reflection (top) and transmission (bottom) configurations. White lines represent the xz intensity profiles passing through the focal point for both operating modes. The right-side insets [(b) and (d)] present the intensity distributions in the xy plane for reflection (top) and transmission (bottom) parts, perpendicular to the optical axis and positioned at the focal point of the structure; Figure S7: Numerical simulation results of two designed metaelements forming Gaussian (a) and Bessel (b) beams in reflection and transmission modes. Numerical simulations predict a DOF of 17.9 mm in transmission and 19.6 mm in reflection, and FWHM of 2.6 mm in transmission and 2.4 mm in reflection for the Gaussian structure, while DOF of 33.3 mm in transmission and 33.8 mm in reflection and FWHM of 2.8 mm in transmission and 3.2 mm in reflection for the Bessel beam-forming structure.

## References

[1] Nagatsuma T. (2011). Terahertz technologies: Present and future. IEICE Electron. Express.

[2] Nagatsuma T., Ducournau G., Renaud C.C. (2016). Advances in terahertz communications accelerated by photonics. Nat. Photonics.

[3] Cui H., Yao J., Wan C. (2011). The study on THz wave propagation feature in atmosphere. J. Phys. Conf. Ser..

[4] Akyildiz I.F., Jornet J.M., Han C. (2014). Terahertz band: Next frontier for wireless communications. Phys. Commun..

[5] Petrov V., Bodet D., Singh A. (2023). Mobile near-field terahertz communications for 6G and 7G networks: Research challenges. Front. Commun. Netw..

[6] Khawaja W., Ozdemir O., Yapici Y., Erden F., Guvenc I. (2020). Coverage Enhancement for NLOS mmWave Links Using Passive Reflectors. IEEE Open J. Commun. Soc..

[7] Chen Z., Ma X., Han C., Wen Q. (2021). Towards intelligent reflecting surface-empowered 6G terahertz communications: A survey. China Commun..

[8] Ma J., Shrestha R., Zhang W., Moeller L., Mittleman D.M. (2019). Terahertz Wireless Links Using Diffuse Scattering From Rough Surfaces. IEEE Trans. Terahertz Sci. Technol..

[9] Bayraktar O., Civi O.A., Akin T. (2012). Beam Switching Reflectarray Monolithically Integrated With RF MEMS Switches. IEEE Trans. Antennas Propag..

[10] Minkevičius L., Indrišiūnas S., Šniaukas R., Voisiat B., Janonis V., Tamošiūnas V., Kašalynas I., Račiukaitis G., Valušis G. (2017). Terahertz multilevel phase Fresnel lenses fabricated by laser patterning of silicon. Opt. Lett..

[11] Driukas S., Ivaškevičiūtė-Povilauskienė R., Čižas V., Redeckas K., Pashnev D., Labanauskas L., Nacius E., Minkevičius L., Valušis G. (2025). C-shaped conducting polymer metalenses fabricated on polyester substrates for focusing terahertz light. Opt. Mater. Express.

[12] Ma X., Chen Z., Chen W., Chi Y., Li Z., Han C., Wen Q. (2020). Intelligent reflecting surface-enhanced indoor terahertz communication systems. Nano Commun. Netw..

[13] Zhuang X., Zhang W., Wang K., Gu Y., An Y., Zhang X., Gu J., Luo D., Han J., Zhang W. (2023). Active terahertz beam steering based on mechanical deformation of liquid crystal elastomer metasurface. Light Sci. Appl..

[14] Tamosiunaite M., Indrisiunas S., Tamosiunas V., Minkevicius L., Urbanowicz A., Raciukaitis G., Kasalynas I., Valusis G. (2018). Focusing of Terahertz Radiation With Laser-Ablated Antireflective Structures. IEEE Trans. Terahertz Sci. Technol..

[15] Siemion A. (2019). Terahertz Diffractive Optics—Smart Control over Radiation. J. Infrared Millim. Terahertz Waves.

[16] Choi M., Lee S.H., Kim Y., Kang S.B., Shin J., Kwak M.H., Kang K.Y., Lee Y.H., Park N., Min B. (2011). A terahertz metamaterial with unnaturally high refractive index. Nature.

[17] Chen J., Hu S., Zhu S., Li T. (2022). Metamaterials: From fundamental physics to intelligent design. Interdiscip. Mater..

[18] Al-Naib I., Withayachumnankul W. (2017). Recent Progress in Terahertz Metasurfaces. J. Infrared Millim. Terahertz Waves.

[19] Li B., Li X., Zhao R., Wang G., Han W., Zhao B., Huang L., Zhang Y., Lu Y., Jiang L. (2020). Polarization Multiplexing Terahertz Metasurfaces through Spatial Femtosecond Laser-Shaping Fabrication. Adv. Opt. Mater..

[20] Yao J., Wang Z., Fan Y., Zhang K., Lin R., Wu J., Peng C., Xiao S., Tsai D.P. (2026). Intensity-asymmetric wavefront shaping in nonlocal meta-lens. Nat. Commun..

[21] Xiao Q., Fan L.H., Ma Q., Ning Y.M., Gu Z., Chen L., Li L., You J.W., Niu Y.F., Cui T.J. (2025). Secure wireless communication of brain–computer interface and mind control of smart devices enabled by space-time-coding metasurface. Nat. Commun..

[22] Zhang J.C., Tsai D.P., Pang S.W. (2026). Non-local bound states in the continuum for nanoscale alignment. Nat. Photonics.

[23] Che X., Liu X., Zhang X., Lei Y., Yu Y., Wang L., Zhou J., Gao Z., Tsai D.P. (2026). Varifocal Alvarez metalens array for adaptive light-field imaging. Nat. Commun..

[24] Yi H., Qu S.W., Ng K.B., Wong C.K., Chan C.H. (2018). Terahertz Wavefront Control on Both Sides of the Cascaded Metasurfaces. IEEE Trans. Antennas Propag..

[25] Zhao R., Zhu Z., Dong G., Lv T., Li Y., Guan C., Shi J., Zhang H. (2019). High-efficiency Huygens’ metasurface for terahertz wave manipulation. Opt. Lett..

[26] Ran J., Xie M., Wen D., Zhang X., Xue C. (2022). Broadband and Dual-Polarized Terahertz Wave Anomalous Refraction Based on a Huygens’ Metasurface. Front. Mater..

[27] Jiang Z., Ding G., Luo X., Wang S. (2024). Photoreconfigurable Metasurface for Independent Full-Space Control of Terahertz Waves. Sensors.

[28] Falcone F., Lopetegi T., Laso M.A.G., Baena J.D., Bonache J., Beruete M., Marqués R., Martín F., Sorolla M. (2004). Babinet Principle Applied to the Design of Metasurfaces and Metamaterials. Phys. Rev. Lett..

[29] Hesmer F., Tatartschuk E., Zhuromskyy O., Radkovskaya A.A., Shamonin M., Hao T., Stevens C.J., Faulkner G., Edwards D.J., Shamonina E. (2007). Coupling mechanisms for split ring resonators: Theory and experiment. Phys. Status Solidi B.

[30] Song Z., Zhao Z., Zhao H., Peng W., He X., Shi W. (2015). Teeter-totter effect of terahertz dual modes in C-shaped complementary split-ring resonators. J. Appl. Phys..

[31] Zhang X., Tian Z., Yue W., Gu J., Zhang S., Han J., Zhang W. (2013). Broadband Terahertz Wave Deflection Based on C-shape Complex Metamaterials with Phase Discontinuities. Adv. Mater..

[32] Ivaškevičiūtė-Povilauskienė R., Čižas V., Nacius E., Grigelionis I., Redeckas K., Bernatonis M., Orlov S., Valušis G., Minkevičius L. (2023). Flexible terahertz optics: Light beam profile engineering via C-shaped metallic metasurface. Front. Phys..

[33] Cizas V., Redeckas K., Stanaitis K., Bieleviciute A., Ivaskeviciute-Povilauskiene R., Jokubauskis D., Karaliunas M., Nacius E., Grigelionis I., Minkevicius L. (2025). Simultaneous Transmission and Reflection Terahertz Homodyne Imaging System with Integrated Resonant C-Shaped Metalenses. ACS Photonics.

[34] Čižas V., Redeckas K., Stanaitis K., Bielevičiūtė A., Ivaškevičiūtė-Povilauskienė R., Grigelionis I., Minkevičius L. (2025). Enhanced dual-mode THz homodyne imaging system with C-shaped metalenses. Proceedings of the Terahertz, RF, Millimeter, and Submillimeter-Wave Technology and Applications XVIII.

[35] Durnin J. (1987). Exact solutions for nondiffracting beams I The scalar theory. J. Opt. Soc. Am. A.

[36] Rao A.S. (2024). A conceptual review on Bessel beams. Phys. Scr..

[37] Huang C., Zappone A., Alexandropoulos G.C., Debbah M., Yuen C. (2019). Reconfigurable Intelligent Surfaces for Energy Efficiency in Wireless Communication. IEEE Trans. Wirel. Commun..

[38] Basar E. (2023). Reconfigurable intelligent surface-empowered MIMO systems. Natl. Sci. Rev..

[39] Batra A., Alam J., Wiemeler M., Goehringer D., Kaiser T. (2021). Long-Range Non-Line-of-Sight THz Sensing. Proceedings of the 2021 46th International Conference on Infrared, Millimeter and Terahertz Waves (IRMMW-THz).

[40] Gomez-Ponce J., Abbasi N.A., Kondaveti R., Kumar A., Abu-Surra S., Xu G., Zhang C., Molisch A.F. (2022). Impact of Common Reflecting and Absorbing Building Materials on THz Multipath Channels. Radio Sci..

[41] Wang Y., Kumar K., Wang L., Zhang X. (2012). Monolithic integration of binary-phase Fresnel zone plate objectives on 2-axis scanning micromirrors for compact microscopes. Opt. Express.

[42] Billuart J., Héron S., Loiseaux B., Amra C., Lequime M. (2021). Towards a metasurface adapted to hyperspectral imaging applications: From subwavelength design to definition of optical properties. Opt. Express.

[43] Yu X., Qian J., Rao D., Wu J., Yang D., Qi D., Wang G., Liu L., Zang X., Han J. (2024). Facile fabrication of THz metasurfaces by a spatially shaped femtosecond laser printing system. Opt. Express.

[44] Sydoruk O., Tatartschuk E., Shamonina E., Solymar L. (2009). Analytical formulation for the resonant frequency of split rings. J. Appl. Phys..

[45] Kumar G., Cui A., Pandey S., Nahata A. (2011). Planar terahertz waveguides based on complementary split-ring resonators. Opt. Express.

[46] Stanaitis K., Čižas V., Bielevičiūtė A., Grigelionis I., Minkevičius L. (2024). High-Impact Polystyrene Structured Light Components for Terahertz Imaging Applications. Sensors.

[47] Feth N., König M., Husnik M., Stannigel K., Niegemann J., Busch K., Wegener M., Linden S. (2010). Electromagnetic interaction of split-ring resonators: The role of separation and relative orientation. Opt. Express.

[48] Zuffanelli S., Zamora G., Aguila P., Paredes F., Martin F., Bonache J. (2015). On the Radiation Properties of Split-Ring Resonators (SRRs) at the Second Resonance. IEEE Trans. Microw. Theory Tech..

[49] Cesiul A., Ikamas K., But D.B., Morkūnaitė I., Lisauskas T., Lisauskas A. (2022). Towards wireless data transmission with compact all-electronic THz source and detector system. Lith. J. Phys..

[50] But D.B., Chernyadiev A.V., Ikamas K., Kołaciński C., Krysl A., Roskos H.G., Knap W., Lisauskas A. (2023). Compact terahertz devices based on silicon in CMOS and BiCMOS technologies. Opto-Electron. Rev..

[51] Ikamas K., Cibiraite D., Lisauskas A., Bauer M., Krozer V., Roskos H.G. (2018). Broadband terahertz power detectors based on 90-nm silicon CMOS transistors with flat responsivity up to 2.2 THz. IEEE Electron Device Lett..

[52] Bitman A., Moshe I., Zalevsky Z. (2012). Improving depth-of-field in broadband THz beams using nondiffractive Bessel beams. Opt. Lett..

[53] Orlov S., Stanaitis K., Kizevičius P., Šlevas P., Nacius E., Minkevičius L., Valušis G. (2025). Single-pixel terahertz imaging with enhanced edge detection using angular momentum of structured light. APL Photonics.

