# Supplementary material for: Reflective and Transmissive Off-Axis Sub-THz Beam Shaping Using Passive Complementary Split-Ring Resonators
Source: Sensors (Basel). 2026 Nov 6;26(21):7013. doi: 10.3390/s26217013 (PMC13766397; doi:10.3390/s26217013)
Supplement: Supplementary file 1 [file sensors-26-07013-s001.zip › sensors-4612981-supplementary.pdf]

Supplementary Material: Reflective and Transmissive Off-Axis Sub-THz Beam Shaping Using Passive Complementary Split-Ring Resonators

Ernestas Nacius, Vladislovas Čižas, Kasparas Stanaitis, Ignas Grigelionis, and Linas Minkevičius[a)]

Department of Optoelectronics, Center for Physical Sciences and Technology, Saulėtekio ave. 3, Vilnius LT–10257, Lithuania

[a)]Electronic mail: linas.minkevicius@ftmc.lt

I. ON THE MATTER OF SIMULATED FOCAL ZONE INTENSITY DISTRIBUTION BY VARIOUS TILT ANGLES

Focal line and perpendicular intensity cuts of numerically simulated fields at the highest intensity points with varying tilting angles from 15° to 75° are depicted in Fig. S1. The simulated depth of focus (DOF), full width at half maximum (FWHM), peak intensity and intensity integrated over the half-maximum area of each focus are presented in Table S1.

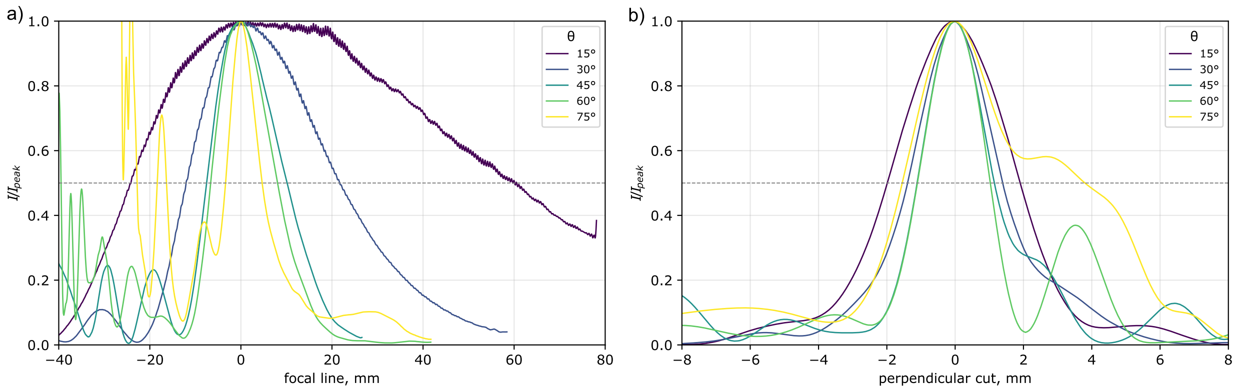

FIGURE S1: Axial intensity profiles (a) along focal line and (b) in perpendicular axis for the Gaussian beam shaping structures, designed to focus radiation at different tilting angles from 15° to 75°. The intensity curves are normalized to their respective maximum values.

Figure S1 Alt text: Two graphs compare the normalized intensity profiles produced by Gaussian beam-shaping structures designed to focus 253 GHz radiation at tilt angles ranging from 15 degrees to 75 degrees. Panel (a) presents the axial intensity profiles measured along the tilted focal line, showing the longitudinal extent and position of the focal region for each designed tilt angle. Panel (b) presents the corresponding transverse intensity profiles measured along an axis perpendicular to the focal line, allowing comparison of the focal-spot widths produced at the different tilt angles. In both panels, each intensity curve is normalized independently to its respective maximum value. The plots therefore compare the shapes, widths, and spatial extents of the intensity distributions rather than their absolute peak intensities.

**Tilt angle**	**DOF, mm (λ)**	**FWHM, mm (λ)**	I_{peak}, **V^2/m^2**	$A_{1/2}$, **mm^2**	$\int_{A_{1/2}} I\,\mathrm{d}A$, **(V/m)^2 mm^2**
**15°**	83.9 (70.5)	4.4 (3.7)	2.75	282.3	559.1
**30°**	33.7 (28.3)	3.2 (2.7)	5.21	73.9	277.1
**45°**	17.9 (15.0)	2.6 (2.2)	0.196	30.9	4.41
**60°**	14.7 (12.3)	2.4 (2.0)	0.172	25.2	3.17
**75°**	8.0 (6.7)	6.1 (5.1)	0.079	37.3	1.90

TABLE S1: Parameters of the simulated transmitted focal zones for tilt angles from 15° to 75°: depth of focus (DOF, full width at half maximum of the intensity along the tilted focal line), FWHM perpendicular to the focal line, peak intensity $I_{\text{peak}} = |E|^2$ for an incident plane-wave amplitude of 1 V/m, area $A_{1/2}$ enclosed by the half-maximum contour of the focus in the xz plane, and intensity integrated over this area. Values in brackets are normalized to the wavelength ($\lambda \approx 1.19$ mm).

Table summarizing the simulated focal zones of Gaussian beam-shaping structures designed for tilt angles from 15 to 75 degrees. For each tilt angle, it lists the depth of focus, defined as the full width at half maximum of the intensity along the tilted focal line; the full width at half maximum of the focal spot perpendicular to the focal line; the peak intensity; the area enclosed by the half-maximum contour of the focus in the xz plane; and the intensity integrated over this area. Lengths are given in millimetres and as multiples of the wavelength of the 253 GHz radiation ($\approx$ 1.19 mm). The peak intensity is highest at 30°, while the integrated intensity decreases monotonically with increasing tilt angle. This sharp decrease is most likely caused by the reduced width of the Fresnel sub-zones, which increases the number of meta-atoms lacking neighbours. This alters their spectral properties and shifts their resonant frequency.

II. ON THE MATTER OF FABRICATION BY LASER ABLATION

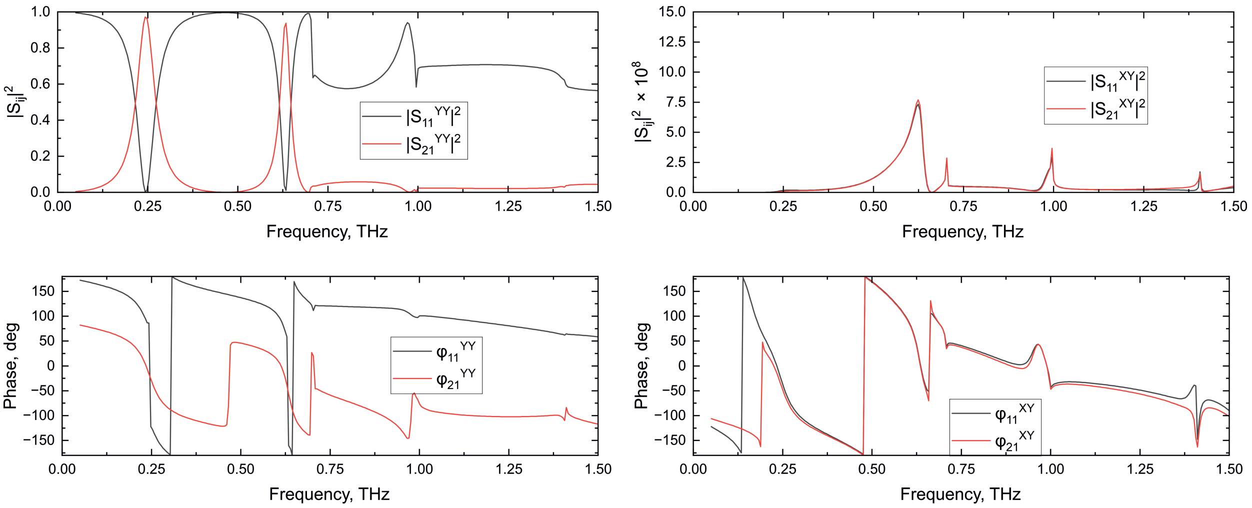

FIGURE S2: Power reflection and transmission (top row) and the corresponding phase spectra (bottom row) of the metaatom with the geometrical parameters used in the manuscript, for y-polarized incidence. Left column: co-polarized response (YY); right column: cross-polarized response (XY). Subscripts denote reflection (11) or transmission (21), and superscripts denote the output and input polarization, respectively.

Spectral parameters of the metaatoms selected for patterning are presented at Figure S2. As expected no perpendicularly excited component exist. Spectral peak position is deliberately chosen to be a bit off the frequency of interest in order to avoid the steep variation of the phase shift near resonance, where small fabrication-induced deviations between nominally identical metaatoms could produce phase mismatch and thereby reduce focusing efficiency. To manufacture surface-based structures and steel foil of 300 µm was used for the Fresnel-like structures. The aim for the manufacturing material and parameter optimization was to acquire high quality CSRR metaatom shape under sufficiently short processing time for the case of metasurface-based structure and to achieve structural rigidity for the Fresnel-like structures. Both structures were cut-through via repetitive laser ablation along a predefined contours. For the final ablation, experimentally optimized processing parameters were defined using the shortest available pulse duration of 290 fs, together with a repetition rate of 50 kHz, pulse overlap of 75% at an average power of 0.45 W. These settings correspond to a scanning speed of 250 mm/s and an energy fluence of 6.8 J/cm^2 per pulse. To achieve cutting through, repeated scanning along the contours was performed at least 120 times. Microscope images of the experimentally cut split-ring resonators are shown in Figure S3.

The manually applied measurements in several areas of the metaelements indicate average values of outer radius $R_{\text{out}} = 168.4 \pm 3$ μm, split-ring thickness $c = 90.5 \pm 3$ μm, and conductive gap angle $\theta = 65.3 \pm 2°$.

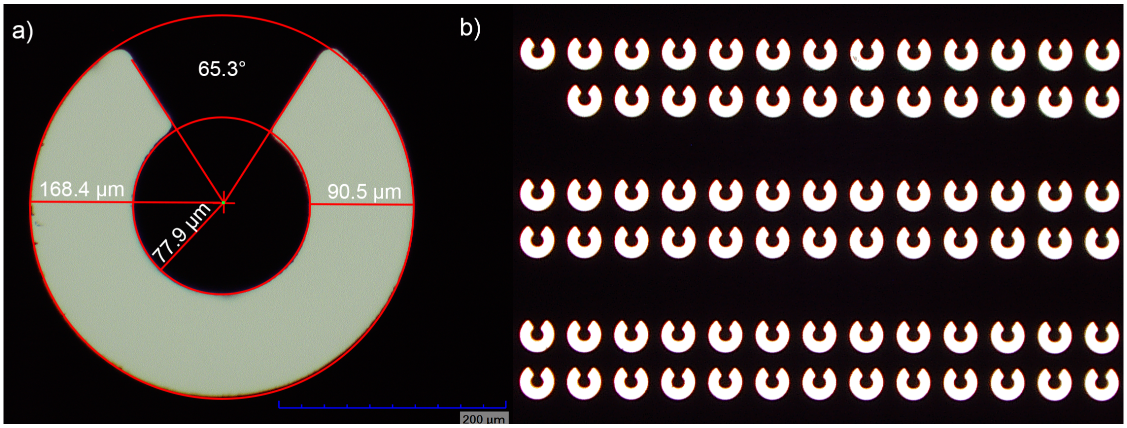

FIGURE S3: Microscope images under backlight illumination of complementary split-ring resonators, cut out by using laser ablation through 25 μm thickness steel foil. (a) shows a single complementary split-ring resonator with measured dimensions: inner and outer radii, cut out angle and ring thickness. (b) shows array of such resonators in reduced magnification, displaying consistency of fabricated structures.

III. ON THE MATTER OF THE EMPLOYED OF PRINCIPAL EXPERIMENTAL SETUP

The principal operating experimental scheme is depicted in Figure S4(a). The THz irradiation detector was mounted on motorized 3D (*xyz*) translation stages with a spherical silicon lens was attached in front for the collection of the irradiation at angle of 45°. The described tilt angle is required to ensure that the emitted radiation reaches the detector without being obstructed by the casing and registering maximum intensity. The correct relative detector tilt angle to the structured focal line of the THz beam was found experimentally at the highest signal level to compensate for lateral beam walk-off [see Figure S4(b)]. Since the electric field in the experiment is oriented along the y axis, this rotation does not affect the measured electric field component at the detector. The fabricated structures are illuminated by a collimated THz source of 253 GHz, ensured by a parabolic mirror. The detector is placed on the xyz translation stages normal to the beam path to scan radiation intensity field both in transmission and reflection regimes. The source and detector employed in the characterization setups are complementary metal–oxide–semiconductor (CMOS) transistor-based THz oscillators[1,2]. The active device substrate, incorporating an integrated log-spiral THz antenna-coupled nanometric field-effect transistor fabricated using 90 nm CMOS foundry technology, is coupled to a 12 mm diameter high-resistivity silicon lens. The devices exhibit a relatively flat response over the 0.1 THz–1.5 THz frequency range[3].

In reflection experiment part, additional absorbers were used during the experiment to reduce reflections thus reducing standing waves, resulting in clean and undistorted measured field patterns. The experimental results show a partially scanned yz distribution due to the limited distance between the focal zone position in space and the detector. The detector structure extends up to 5 cm, which physically restricted the distance and prevented a full field scan.

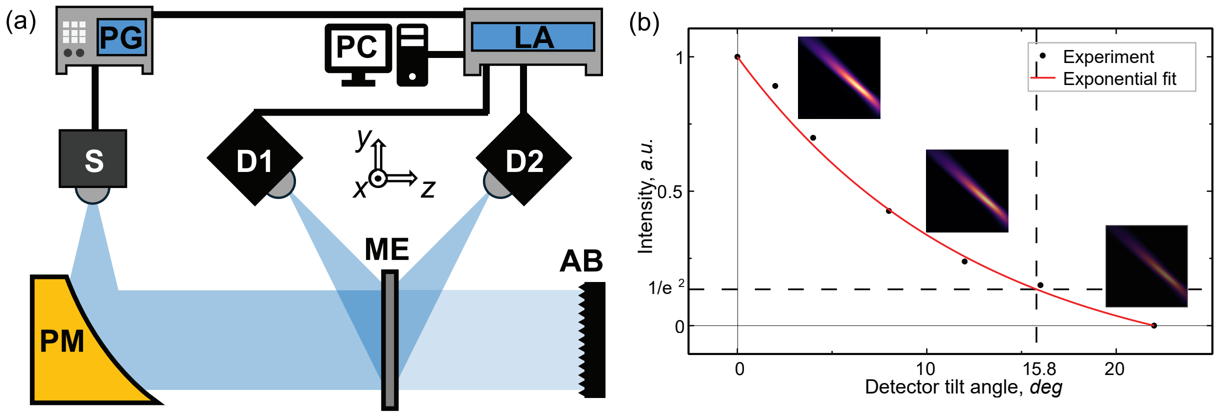

FIGURE S4: (a) The experimental scheme for the investigation of the dual-mode off-axis beam-shapers: PG – pulse generator "Agilent 33519B", LA – Lock-in amplifier "7265 DSP Signal Recovery", S – linear polarization source "CMOS Colpitts", emitting 78 µW power 253 GHz radiation, PM - parabolic mirror of focal length $f = 15$ cm, PC – computer used for process automatization and a 90 nm CMOS TeraFET detector synchronous scanning in *xyz* axes (*y* – LTS300C, *xz* – DDS300, both products of *ThorLabs, Inc.*), D1 and D2 represent two different positions in which a THz detector was placed during scans. AB - radiation absorber, which ensures no back-reflections and detector biases occur. (b) Angular dependence of the normalized focal spot signal measured with the lens-coupled CMOS TeraFET detector module, where black markers indicate experiment, red curve is exponential fit. The dashed horizontal line indicates the $1/e^2$ level and the vertical dashed line marks the corresponding characteristic angle ($\alpha_{1/e^2} = 15.8°$). The insets show representative measured focal spot intensity maps at selected tilt angles.

Figure S4 Alt text: Two-panel figure presenting the experimental arrangement and the measured angular response of the dual-mode off-axis beam shapers. Panel (a) is a schematic of the terahertz measurement setup. A pulse generator provides the modulation signal, and a Signal Recovery lock-in amplifier records the detector r esponse. A linearly polarized source emits 78 microwatts of radiation at 253 GHz. The radiation is directed by a parabolic mirror with a focal length of 15 cm toward the beam-shaping structure. A detector is translated synchronously along the x, y, and z axes using motorized stages, while a computer controls and automates the scanning process. Labels D1 and D2 indicate the two detector positions used for the measurements. A radiation absorber is positioned to suppress unwanted back-reflections and prevent associated detector-bias effects. Panel (b) shows the normalized focal-spot signal measured with the lens-coupled detector

as a function of tilt angle. Black markers represent the experimental measurements, and a red curve represents an exponential fit to the measured angular dependence. A horizontal dashed line identifies the intensity level equal to $1/e^2$ of the maximum signal. A vertical dashed line marks the corresponding characteristic angle, which is 15.8 degrees. Insets display representative two-dimensional focal-spot intensity maps measured at selected tilt angles, illustrating how the detected focal distribution changes with the angular position.

Figure S5 reveals intensity profile acquired after the parabolic mirror of the setup. It is clearly visible, the profile is not ideally uniform: FWHM of 12.3 mm and 15.8 mm in x and y axes, respectively have been acquired. While such non-uniformity does not affect the on-axis performance of the metasurface, the off-axis performance can be significantly influenced as is revealed in the analysis of the Gaussian beam forming structures.

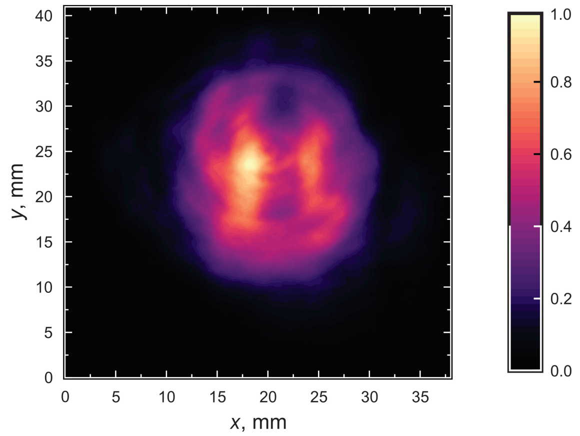

FIGURE S5: Intensity profile, acquired after the parabolic mirror in the setup. Note the non-uniformity in the profile, explaining discrepancies of the simulated and experimentally achieved results.

Figure S5 Alt text: Two-dimensional measured intensity distribution of the 253 GHz beam acquired after propagation through the parabolic mirror used in the experimental setup. The map shows that the incident radiation is spatially non-uniform rather than having an ideal, uniform, or perfectly symmetric beam profile. Variations in intensity are present across the measured area, indicating that different regions of the beam illuminate the beam-shaping structure with different incident intensities. This non-uniform illumination provides a possible explanation for differences between the numerically simulated focal distributions, which assume idealized illumination conditions, and the focal distributions obtained experimentally.

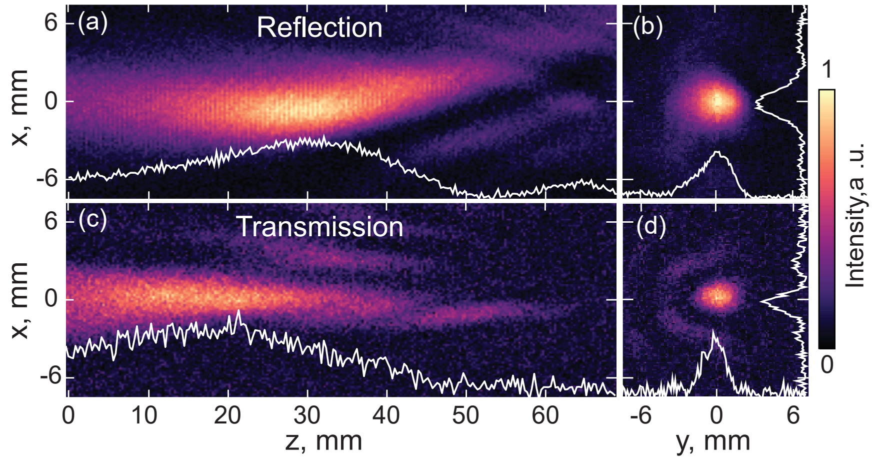

FIGURE S6: Characterization of the Fresnel lens-based beam shaper operating in reflection and transmission modes. Figures on the left [(a) and (c)] show the intensity distributions in the *xz* plane corresponding to the focal region of the structure for reflection (top) and transmission (bottom) configurations. White lines represent the *xz* intensity profiles passing through the focal point for both operating modes. The right-side insets [(b) and (d)] present the intensity distributions in the *xy* plane for reflection (top) and transmission (bottom) parts, perpendicular to the optical axis and positioned at the focal point of the structure.

IV. ELECTRIC FIELD DISTRIBUTION SHAPED BY ELLIPTICAL FRESNEL REFLECTOR

The experimental configuration enabled conventional transmissive and reflective beam-shaping characterization[4,5]. Spatial scanning was performed in two orthogonal planes: the *xy* plane, defined perpendicular to the optical axis at the focal position, and the *xz* plane, aligned parallel to the optical axis. The acquired *xz* intensity distribution and the corresponding intensity profiles passing through the focal point are shown in Figure S6 with reflected and transmitted beams provided. The measurements were performed using pixel size of 15 µm × 15 µm. For the Fresnel lens-based structure, the full width at half maximum (FWHM) of 3.7 mm ($\approx 3.1\lambda$) and the depth of field (DOF) of 27.3 mm ($\approx 22.9\lambda$) were obtained in the reflection mode and the FWHM of 2.1 mm ($\approx 3.5\lambda$) and the DOF of 26 mm ($\approx 21.8\lambda$) in the transmission. The comparatively large DOF is characteristic for the diffractive-optics-based beam-shaping elements and is consistent with previously reported results in the literature cited above.

Figure S6 Alt text: Four intensity maps characterize the operation of the Fresnel lens-based

beam shaper in reflection and transmission configurations. The two maps on the left show intensity distributions in the xz plane, which contains the optical axis and passes through the focal region. Panel (a) presents the xz-plane distribution for the reflected component of the radiation, while panel (c) presents the corresponding distribution for the transmitted component. These longitudinal maps show the location, shape, and spatial extent of the focal regions formed in the two operating modes. A white line in each xz map indicates the path through the focal point along which the corresponding axial intensity profile is evaluated. The two maps on the right show transverse intensity distributions in the xy plane, positioned perpendicular to the optical axis at the respective focal locations. Panel (b) presents the focal-plane intensity distribution for the reflected radiation, and panel (d) presents the focal-plane intensity distribution for the transmitted radiation. Together, the longitudinal and transverse maps provide complementary views of the focal regions and allow the focusing performance of the Fresnel lens-based beam shaper to be compared between its reflection and transmission modes.

V. NUMERICALLY SIMULATED FIELDS IN TRANSMISSION AND REFLECTION FOR THE GAUSSIAN AND BESSEL METASURFACES

Figure S7 Alt text: Numerical simulation results for two metasurface-based structure designs that generate different beam types while operating simultaneously in reflection and transmission modes. Panel (a) presents the simulated intensity distributions produced by the metastructure designed to form a Gaussian beam. Panel (b) presents the simulated intensity distributions produced by the metastructure designed to form a Bessel beam. Comparison of the two designs shows that the simulated Bessel beam has a substantially longer depth of field than the Gaussian beam, while also having a somewhat larger transverse full width at half maximum.

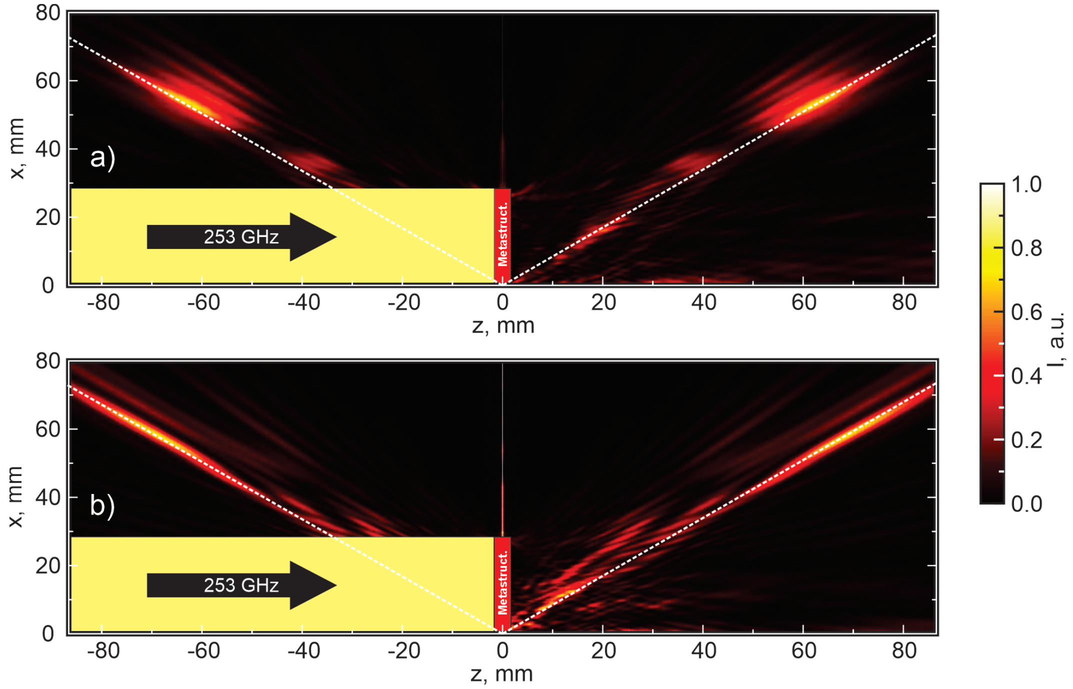

FIGURE S7: Numerical simulation results of two designed metaelements forming Gaussian (a) and Bessel (b) beams in reflection and transmission modes. Numerical simulations predict a DOF of 17.9 mm in transmission and 19.6 mm in reflection and FWHM of 2.6 mm in transmission and 2.4 mm in reflection for Gaussian structure, while DOF of 33.3 mm in transmission and 33.8 mm in reflection and FWHM of 2.8 mm in transmission and 3.2 mm in reflection for Bessel beam forming structure.

REFERENCES

[1] A. Cesiul, K. Ikamas, D. B. But, I. Morkūnaitė, T. Lisauskas, and A. Lisauskas, "Towards wireless data transmission with compact all-electronic thz source and detector system," Lithuanian Journal of Physics **62** (2022).

[2] D. B. But, A. V. Chernyadiev, K. Ikamas, C. Kołaciński, A. Krysl, H. G. Roskos, W. Knap, and A. Lisauskas, "Compact terahertz devices based on silicon in cmos and bicmos technologies," Opto-electronics review. **31**, 1–10 (2023).

[3] K. Ikamas, D. Čibiraitė, A. Lisauskas, M. Bauer, V. Krozer, and H. G. Roskos, "Broadband terahertz power detectors based on 90-nm silicon cmos transistors with flat responsivity up to 2.2 thz," IEEE Electron Device Letters **39**, 1413–1416 (2018).

[4]R. Ivaškevičiūtė-Povilauskienė, V. Čižas, E. Nacius, I. Grigelionis, K. Redeckas, M. Bernatonis, S. Orlov, G. Valušis, and L. Minkevičius, "Flexible terahertz optics: light beam profile engineering via c-shaped metallic metasurface," Frontiers in Physics **11** (2023), 10.3389/fphy.2023.1196726.

[5]V. Cizas, K. Redeckas, K. Stanaitis, A. Bieleviciute, R. Ivaskeviciute-Povilauskiene, D. Jokubauskis, M. Karaliunas, E. Nacius, I. Grigelionis, and L. Minkevicius, "Simultaneous transmission and reflection terahertz homodyne imaging system with integrated resonant c-shaped metalenses," ACS Photonics **12**, 636–647 (2025).
